# Advanced Hydrogels in Fibrocartilage Regeneration of the Glenoid Labrum

**DOI:** 10.3390/gels11080652

**Published:** 2025-08-18

**Authors:** Benjamin R. Caruso, Jihun Cha, Tammam Hanna

**Affiliations:** 1 Elson S. Floyd College of Medicine, Washington State University, Spokane, WA 99202, USA; jihun.cha@wsu.edu; 2 Department of Orthopaedic Surgery and Rehabilitation, Texas Tech University Health Sciences Center, Lubbock, TX 79430, USA; tammam.hanna@ttuhsc.edu

**Keywords:** hydrogel, tissue engineering, cartilage regeneration, regenerative medicine, drug delivery, labral injury

## Abstract

Glenoid labral tears are relatively common orthopedic injuries in adults. Anatomically, the glenoid labrum is a fibrocartilaginous structure that contributes to shoulder stability and function. The treatment for labral injury may be conservative, such as activity modification and rest, or operative, depending on the extent of tissue damage. Hydrogels are polymeric networks with great potential in treating glenoid labral tears and other cartilage-related injuries. Hydrogels are highly biocompatible, hydrophilic, and non-immunogenic, with tunable mechanical properties that support nutrient diffusion, cell viability, and angiogenesis, making them well suited for cartilage regeneration. Hydrogels can deliver growth factors like TGF-β or PDGF and may be combined with peptides or adhesion molecules to enhance tissue integration, repair, and even physical support. This article introduces current treatment options for glenoid labral injuries, reviews the role of hydrogels in cartilage regeneration, and summarizes recent translational research focused on hydrogel-based labral repair.

## 1. Introduction

Glenoid labral tears are relatively common, with a prevalence of 35% among athletes and 6% in the general population [1,2]. Patients most likely sustain glenoid labral tears during repetitive overhead activities and traumatic shoulder dislocation or subluxation [3,4]. Thus, the populations most commonly affected by glenoid labral tears are overhead athletes and older adults, often following traumatic injury to a degenerating and aging labrum [3,4,5]. The most frequently encountered labral tear is the anteroinferior Bankart lesion, followed by superior labral anterior to posterior (SLAP) tears. Less common injuries include anterior labral periosteal sleeve avulsion (ALPSA) lesions and glenoid internal rotation deficit (GIRD) [6].

The glenoid labrum is a fibrocartilaginous structure that forms a rim around the glenoid cavity, commonly referred to as the socket of the shoulder [7,8]. The dense cartilaginous matrix is built and maintained by fibrochondrocytes. Fibrochondrocytes synthesize the cartilaginous matrix through a diverse and complex network of growth factors and chemical signaling molecules. These include platelet-derived growth factor (PDGF), insulin-like growth factor 1 (IGF-1), transforming growth factor beta (TGF-β), fibroblast growth factors (FGF), and others [9,10]. Additionally, fibrochondrocytes are mechanosensitive, altering extracellular matrix synthesis in response to physical stimuli such as shear stress, compressive forces, and cyclic loading [10,11,12,13]. Fibrochondrocytes synthesize both type I and type II collagen, as well as proteoglycans, producing an extracellular matrix that provides both tensile strength and elasticity to the glenohumeral joint [14,15,16].

In addition to enhancing joint stability by forming a ring of cartilage around the humeral head, the labrum also deepens the glenoid cavity. It acts as a cushion, allowing for diverse shoulder joint movement [7,17]. Functionally, the superior labrum serves as an attachment site for the long head of the biceps tendon [7]. The labrum is also essential for joint congruency, proprioception, and force transmission during overhead movements [17,18]. These features make the labrum both protective and structurally critical to the glenohumeral joint’s anatomy and biomechanics.

Tears of the glenoid labrum can be challenging for orthopedic surgeons to manage. Firstly, labral tears often occur with other injuries such as rotator cuff tears and biceps tendinosis [19,20]. Labral tears are not uniform and can involve any portion of the ring-shaped labrum [21], further complicating which treatments are indicated for patients sustaining these injuries. Weakly supplied by the circumflex humeral arteries, the glenoid labrum is known to have limited surrounding vasculature, especially in the superior region [22]. This adds significant challenges in the healing process of glenoid labral tears, as damaged regions often face difficulty in receiving proper nutrients, tissue stimulating growth factors, and blood flow required for sufficient healing [22].

Standard treatment for glenoid labral tears is dependent on the type of tear, severity, and the demographics of the patient [3,23,24]. For minor tears, orthopedic surgeons will often prescribe nonoperative treatments, including limiting activity, physical therapy, corticosteroid injections, and nonsteroidal anti-inflammatory drugs (NSAIDs) to reduce pain and inflammation [23,24]. For significant labral tears or when conservative management fails, operative treatment is often indicated [3,25]. Operative treatment for glenoid labral tears includes arthroscopic labral debridement, arthroscopic labral repair where the labrum is reattached using suture anchors, including Bankart and SLAP repairs, biceps tenodesis, or tenotomy, each selected based on the tear type, patient age, activity level, and associated shoulder pathology to restore joint stability and function [3,4].

The success of glenoid labral tear treatment depends on several factors, including the type and severity of the tear, treatment modality, patient demographics (such as age and BMI), activity level, and adherence to rehabilitation protocols [26,27]. While numerous studies report favorable outcomes with operative management, overall success rates remain suboptimal, with many patients continuing to experience pain, limited function, or failure to return to previous activity levels [28,29]. Conservative treatments tend to show even lower success rates [30,31]. Common complications following surgical repair include arthrofibrosis, retear, and persistent pain [3,32,33], while rarer issues such as infection, nerve injury, and residual laxity may also occur [34,35]. These challenges have driven the pursuit of improved techniques such as knotless anchors, remplissage for concurrent Hill-Sachs lesions [36], and biologic augmentations to enhance fixation and healing outcomes.

**Figure 1:**
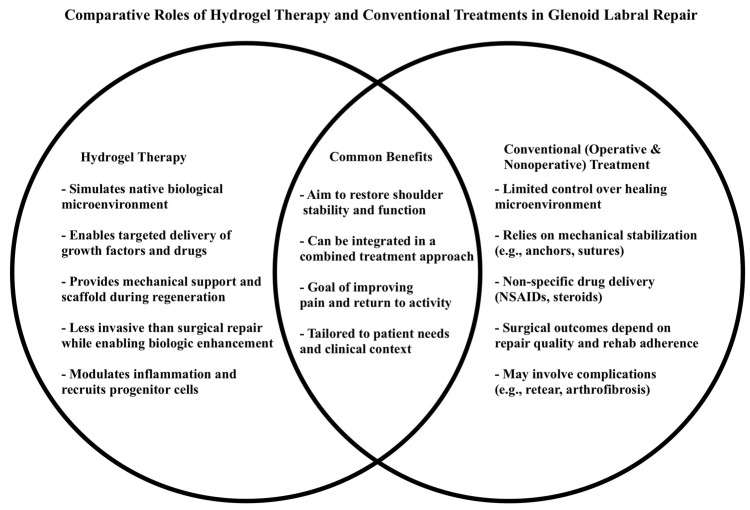
Comparative roles of hydrogel therapy and conventional treatments in glenoid labral repair. Hydrogel-based approaches offer biologically active, regenerative advantages and can be used adjunctively to overcome limitations of traditional operative and nonoperative modalities. Together, these strategies may support improved healing, functional outcomes, and long-term joint preservation.

One technology that displays increasing potential for the treatment of glenoid labral tears and cartilage-related injuries is hydrogels. Hydrogels can be desirable in orthopedically related injuries because of their biomimetic and regenerative properties [37,38]. Some hydrogels are characterized by their high biocompatibility and low immunogenicity, reducing the risk of adverse tissue reactions. Their structures can be precisely engineered to control mechanical strength, degradation rates, and porosity, allowing for close mimicry of the native cartilage environment (Figure 1).

**Figure 2:**
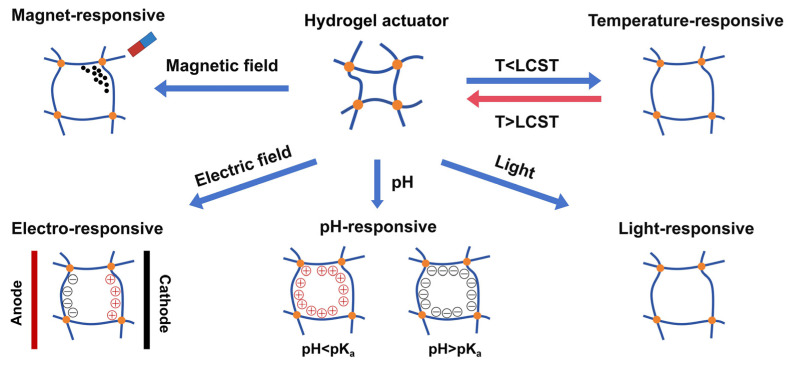
Common stimuli responsive behaviors of hydrogel actuators. Hydrogel networks can be engineered to respond to external triggers such as magnetic fields, temperature changes, electric fields, pH variations, and light exposure. These responsive properties can be leveraged in orthopedic applications, including glenoid labral repair, to enable controlled gelation, targeted drug release, and dynamic mechanical adaptation. Adapted from “Stimuli-responsive hydrogel actuators for skin therapeutics and beyond,” Soft Science, 2024, licensed under CC BY 4.0 [39].

Beyond their fundamental biocompatibility, hydrogels can be engineered to respond to specific environmental stimuli, enabling tailored therapeutic effects. Stimuli such as temperature, pH, magnetic and electric fields, and light can trigger gelation, modulate mechanical properties, or control the release of embedded biologics. These capabilities expand the potential of hydrogels in orthopedic applications, including glenoid labral repair, where on-demand responsiveness may enhance integration, regeneration, and functional outcomes (Figure 2) [39].

In our review, we summarize the most recent advancements in hydrogels in cartilage regeneration with a focus on translational studies of injuries related specifically to the glenoid labrum.

## 2. Advancements in Hydrogels in Cartilage Regeneration and Healing of Soft Tissue Injuries

Because of their biological versatility, hydrogels are increasingly being researched and experimented with in the disciplines of musculoskeletal and soft tissue engineering. Today, hydrogels are commonly used clinically in wound dressings to facilitate drug delivery and healing [40,41]. Similar hydrogels are also being studied for angiogenic support, muscle cell delivery, nerve healing, and antineoplastic therapy [42,43,44,45]. A particularly exciting use of hydrogels is in cartilage regeneration. From 2000 to 2022, 365 clinical trials were registered in cartilage regeneration using biologics, with an observed acceleration in hydrogel use in recent years [46].

Cartilage damage can be managed conservatively with a focus on pain management and rehabilitation. Surgical interventions are considered in severe cases for further relief of symptoms [47,48]. However, these therapy options do not have the ability to regenerate damaged cartilage. In recent years, scaffold materials such as hydrogels with cells or growth factors have shown promise as a method of cartilage regeneration [49,50]. To properly support the repair of cartilage damage, it is crucial for the scaffolds loaded with stem cells and cellular growth factors to resemble natural extracellular matrix structure. Hydrogels are polymer networks with hydrophilic functional groups, allowing them to swell and hold a significant amount of water and exhibit a degree of flexibility similar to native tissues. They also possess resistance to dissolution by water due to the crosslinks between polymer networks [51,52]. Due to their versatility and suitability, the biological application, synthesis, and characterization of hydrogels have been emerging topics in the field of tissue engineering for the past few decades [53,54,55].

### 2.1. Natural and Synthetic Hydrogels in Cartilage Regeneration

Hydrogels can be synthesized by crosslinking polymers. Both naturally occurring or synthetic polymers can be used. Natural hydrogels include natural biomolecules such as collagen, alginate, hyaluronic acid (HA), chitosan, and dextrans [56,57]. These native, abundant materials are commonly used because of the supportive role they play in cartilage repair [50]. Some natural hydrogels also offer an advantage in biocompatibility since they may be used without concerns of immunological responses, whereas synthetic gels may require additional modifications to enhance their biocompatibility [51].

In recent years, natural hydrogels have emerged as a safe and effective delivery system for cells and growth factors to promote cartilage regeneration [58]. A lyophilized gelatin–HA hydrogel successfully regenerated cartilage in vitro, which was subsequently implanted in nude mice and goat models. Engineered cartilage showed continued regeneration of cartilage-like tissue without immunological responses [59]. Chitosan-based hydrogels seeded with chondrocytes have successfully repaired articular cartilage defects in rabbit models, supporting integration with host tissue and promoting hyaline-like cartilage regeneration (Table 1) [60]. This result indicates that natural polymers can undergo preservation processes to overcome the dilemma of mechanical integrity.

Despite high biocompatibility and low cytotoxicity, natural hydrogels lack appropriate physical properties such as mechanical strength [61], resulting in fast degradation and difficulty in maintaining the original three-dimensional shape [59]. This complicates the clinical use of natural hydrogels for cartilage regeneration, since polymers would need to withstand the implantation procedures and postoperative stress. Rapid degradation also poses an issue as the regeneration process requires an optimal degradation rate to maintain integrity while avoiding immunological reactions and maximizing cellular infiltration [57]. Natural polymers are also susceptible to unique challenges such as variation of quality between batches and potential transmission of pathogens or zoonotic diseases from xenogeneic ingredients [62,63,64]. Natural polymers extracted from animal sources are especially impacted by the challenge of batch-to-batch inconsistency. Difficulty related to reproducibility and compositional consistency in biopolymer-based hydrogels have been noted in the literature previously [65].

Synthetic hydrogels can address disadvantages related to strength and rapid degradation. A wide selection of synthetic hydrogels is commercially available for cartilage regeneration. The most widely used synthetic hydrogels include PEG and polyvinyl alcohol (PVA), although other polymers are available, such as polycaprolactone (PCL) (Table 1) [66,67]. Compared to natural hydrogels, gels made of synthetic polymers provide strong mechanical stability and higher capacity for water absorption, which provide adequate flexibility similar to natural extracellular matrix [52,68]. Synthetic gels are also useful due to the relative ease of manipulation and control over mechanical structures if the material is carefully selected to avoid the biocompatibility issue [51]. For example, synthetic hydrogels can be modified to have a desired shape or degradation time through various techniques, including blending with other polymers [66].

Because of numerous advantageous properties of synthetic gels listed above, synthetic hydrogels have been tested previously as a cell and/or drug delivery system in many studies [49]. PEG hydrogel with tethered growth factors resulted in a similar level of cartilage regeneration in vitro and in vivo, though engineered tissues observed in vivo were more extensive and of higher quality [69].

Despite several advantages, clinicians and researchers may face challenges when applying synthetic hydrogels due to several disadvantages of synthetic materials, including lower biocompatibility and the possibility of adverse effects caused by cytotoxicity from sources such as crosslinking agents [68,70]. The obstacle in hydrogel use for cartilage regeneration and their clinical application is that both purely natural and purely synthetic hydrogels come with downsides, emphasizing the need for a strategy to develop novel hydrogels with minimal disadvantages.

### 2.2. Hybrid Hydrogels

Although natural and synthetic polymers by themselves can be effective modalities for cartilage regeneration through different processing techniques, the search for more suitable materials has continued over the past years. The ongoing development of hydrogels with minimal drawbacks is important, especially for cartilage regeneration with hydrogels in the clinical stage. The ideal material should be biocompatible and degrade to nontoxic compounds while providing adequate mechanical strength [66]. Numerous strategies have emerged to combine the advantages of natural and synthetic gel polymers. One such strategy is the combination of natural polymers into complex hydrogel scaffolds to improve strength. Zhao et al. [61] reviewed the use of natural polymers such as chitosan, gelatin, hyaluronic acid, and alginate in hydrogel scaffold design, highlighting their favorable biocompatibility, tunable degradation, and strategies to improve mechanical strength through crosslinking and polymer blending. For example, chitosan–gelatin and silk fibroin–chitosan composites demonstrated enhanced structural integrity and supported chondrocyte proliferation and extracellular matrix production in preclinical models (Table 1) [61].

Another strategy that gained popularity recently is the combination of natural polymers into complex, hybrid hydrogel scaffolds to improve strength. Hybrid hydrogels are fabricated using diverse materials, each exhibiting distinct characteristics. The obvious advantages of hybrid gels include improved mechanical strength and the ability to mimic native extracellular matrices [71]. Polysaccharide-based or peptide-based natural polymers, with their superior biocompatibility and non-toxic biodegradation, can be combined with synthetic polymers such as PEG and PVA to enhance control over degradation and mechanical stability (Table 1) [71,72]. This strategy supplements the limitations of natural and synthetic polymers and strengthens the versatility of hydrogel in biomedical research.

**Figure 3:**
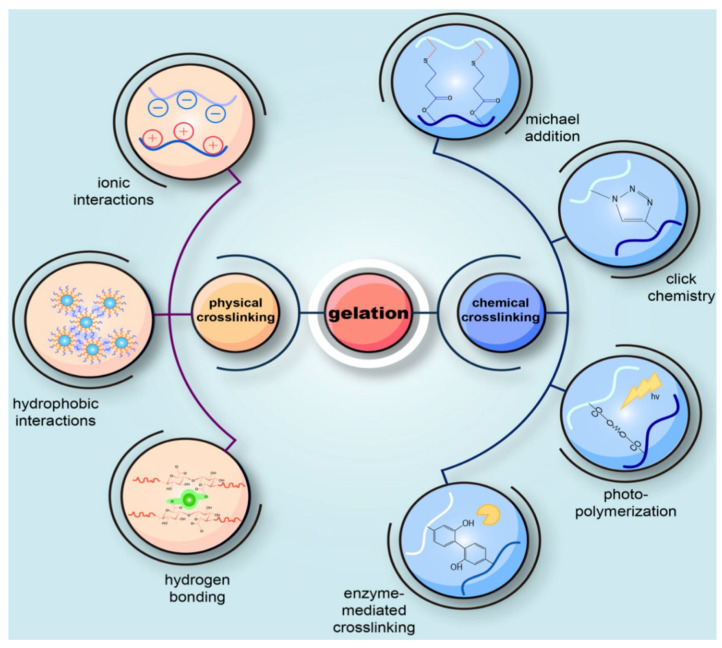
Schematic diagram of common hydrogel crosslinking mechanisms (physical vs. chemical). Reproduced from Wu et al., “Exquisite Design of Hydrogels in Cartilage Repair,” Theranostics 2020; 10, 6864–6892, licensed under CC BY 4.0 [74].

Hybrid hydrogels can be divided into different types based on the method of crosslinking (Figure 3). They can be maintained by physical crosslinking, driven by molecular entanglement and secondary forces such as hydrophobic interactions and hydrogen bonding [73,74]. Physically crosslinked hydrogels are reversible and relatively easy to produce. Chemical crosslinking, on the other hand, yields a more stable hydrogel with covalent bonds. Chemically crosslinked gels can be fabricated through several different methods, including enzymatic reaction and free radical polymerization of monomer units among many others (Table 1) [73,75,76]. Although crosslinking can provide enhancement of mechanical strength and durability through covalent bonds between polymer chains, organic crosslinking agents used in preparation must be removed before use, as residues of these agents are known to cause toxicity in the human body [77]. Past studies have used citric acid as a crosslinking agent to overcome the toxicity issue [78]. Recently, Capanema et al. synthesized carboxymethyl cellulose/PVA hybrid hydrogel using citric acid as a chemical crosslinking agent [79]. Although many challenges still remain, hybrid hydrogels hold great promise in overcoming limitations of natural and synthetic polymers. Various types of hybrid hydrogels, each with distinguishing characteristics, are available for specific applications.

The current literature indicates that the fabrication of hydrogels by combining different polymers can support cellular growth through modulation of the degradation rate while maintaining mechanical stability, biocompatibility, and nontoxicity [51,80]. To achieve clinical application of hydrogels for cartilage regeneration, the enhancement of biological and mechanical properties through the combination of materials remains an important next step [81].

### 2.3. Translational and Preclinical Research

The efficacy of hydrogels in cartilage regeneration has previously been shown in animal models. In vivo injection of hydroxypropyl chitin (HPCH) hydrogel with chondrocytes was demonstrated to be adequate for cartilage regeneration in nude mice models [82]. Intradermal hydrogel releasing HA showed a significant, dose-dependent increase in bone mineral density in osteoarthritic mouse models [83]. Zeng et al. [84] observed in vivo cartilage regeneration in a rat model using pig-derived decellularized extracellular matrix (dECM) hydrogel encapsulating human urine-derived stem cells (USCs). Notably, the dECM hydrogel alone without USCs resulted in the enhancement of repair of full-thickness cartilage defect [84]. In addition to hydrogel’s role in native cartilage regeneration via cell and growth factor, the development of artificial glenoid labrum using synthetic hydrogel has been attempted previously. A study by Wahab et al. [85] showed that PVA hydrogel can be used to replicate the intact native glenoid labrum when it is coupled with multiple cycles of the freeze–thaw method. Notably, the study found that increasing the concentration of PVA correlated with a higher compressive modulus, allowing the hydrogel’s stiffness to be tuned to match that of the native glenoid labrum. The authors elaborated further that PVA-based artificial glenoid labrum is capable of mitigating glenoid component loosening, a common postoperative complication of total shoulder arthroplasty, by reducing the stress applied on the polyethylene implant by up to 51% [85] (Table 2). Although PVA-based artificial glenoid labrum has not been tested for in vivo transplantation and therefore its efficacy and biocompatibility are largely unknown, this highlights yet another potential application of hydrogel in labral injury besides supporting cartilage regeneration.

PEG-based hydrogel was also tested in a large-animal model and a pilot clinical study with human patients. In a caprine model, PEG-based hydrogel in combination with microfracture repair showed a greater filling of chondral defects when compared to microfracture repair alone [86]. A similar result was seen in a human study, where the patients who received hydrogel implants in addition to microfracture obtained a greater degree of defect filling and a significant reduction in pain severity and frequency [86]. These studies suggest that hydrogels with encapsulated cells and/or growth factors hold potential for cartilage regeneration and eventual translation to clinical use.

Labrum chondrogenesis augmentation has been attempted in prior studies. Transplantation of autologous chondrocytes with allogenic flexor digitorum profundus in a rabbit model resulted in increased cartilage cell growth [87]. Although not specific to the labral injury, studies used hydrogels for cartilage regeneration in the setting of rotator cuff injury. Similar to labrum, rotator cuff tendons consist of fibrous cartilage. Delivery of factors such as kartogenin and FGF via chitosan-based hydrogel successfully promoted fibrocartilage formation (Table 2) [88].

**Table 2:** Summary of hydrogel-based platforms evaluated in translational studies for cartilage and labral repair.

Source/Composition	Biologic Additive(s)	Study Model	Target Tissue	Advantages	Disadvantages	Ref.
Porcine SIS ECM + hUSCs	hUSCs overexpressing bFGF	In vivo (TBI model)	Tendon–bone interface	Integrates an immunomodulatory SIS hydrogel with stem cell-based chondrogenesis Improves fibrocartilage regeneration and mechanical strength at the repair site; uses non-invasively obtained hUSCs with high transduction efficiency Enables sustained gene expression and tissue-specific healing via in situ gelation	Lentiviral gene delivery carries a risk of insertional mutagenesis Effects observed only in small-animal (rat) model; limited translation data Immune modulation mechanisms remain incompletely defined Potential oversimplification of macrophage phenotypes (M1 vs. M2 dichotomy) Long-term safety of gene-modified stem cells in humans is unknown	[9]
Chitosan + KGN + FGF-2	KGN and FGF-2	Ex vivo human	Acetabular labrum	Dual delivery of FGF-2 and KGN promotes both stem cell proliferation and chondrogenic differentiation Enhances fibrocartilage regeneration at the tendon–bone interface Sustained release profile improves factor bioavailability during early healing Biodegradable and biocompatible; no adverse effects on surrounding tissues Demonstrates synergistic action of growth factors, improving tendon structural integrity	High concentrations of KGN may induce apoptosis or off-target cartilage formation Lack of mechanistic clarity on FGF-2 and KGN interaction No long-term or large-animal data confirming durability or integration under joint stress Targeted to rotator cuff healing; requires adaptation for glenoid labrum applications	[85]
Chitosan + PDGF	PDGF	Rat model (in vivo)	Glenoid labrum	Targets biologically challenging regions like the superior glenoid labrum Enhances recruitment of progenitor cells (CD44+ and CD73+) Reduces inflammation (e.g., IL-1β and MMP-13 downregulation) Promotes ECM production (collagen and GAGs) Injectable format enables minimally invasive delivery	Evaluated only in a small-animal (rat) model Limited evidence of long-term durability or functional integration Unclear if findings translate to human biomechanical demands No comparative control group or standard-of-care comparator	[88]

The recent emergence and advancement of three-dimensional (3D) bioprinting can potentially be used for developing scaffolds to treat a wide range of orthopedic pathologies such as osteoarthritis [89]. Bio-inks are an integral part of manufacturing scaffolds through bioprinting technology. In general, bio-inks refer to a composition of living cells along with biomaterial to create biological structures [90]. Hydrogel loaded with cells or factors is a common form of bio-ink used to create a natural microenvironment to support cartilage repair and regeneration. Compared to conventional methods, bioprinting comes with several different advantages. The design process in bioprinting can be carried out using software that allows for a design that is specific to each patient [89]. Bioprinting also enables researchers to produce tissues using various combinations of cell types and biomaterials with high reproducibility [91]. Clinical use of cell-laden hydrogels requires a careful evaluation of safety, compatibility, reproducibility, and cost in addition to efficacy. Bioprinting can serve as a useful tool in engineering an ideal material for novel therapies that promote cartilage repair and regeneration. To our knowledge, no prior study about use of bioprinting specific to fibrocartilage regeneration of glenoid labrum has been done to date. This is likely due to recent emergence of bioprinting technology. However, it seems that preclinical and translational studies discussed in this review may also be conducted using bioprinting technology rather than conventional methods. Further studies on bioprinting specific for glenoid labral regeneration are warranted to measure its potential utility.

## 3. Advancements of Hydrogels in Glenoid Labral Tear

Despite the growing interest in hydrogels for cartilage regeneration, their targeted application to glenoid labral injuries remains underexplored. The glenoid labrum poses distinct challenges for regenerative strategies due to its fibrocartilaginous composition, relatively avascular architecture, and mechanically demanding environment. These features contribute to a limited intrinsic healing capacity and a higher likelihood of poor outcomes following both conservative and surgical interventions. As a result, recent efforts in regenerative medicine have focused on enhancing the biological function of hydrogels through the incorporation of therapeutic additives that can modulate the microenvironment and stimulate tissue repair.

While most of the current data originate from studies in general cartilage repair or hip labral models, the underlying principles are highly translatable to glenoid pathology. The incorporation of various growth factors and drugs into hydrogel platforms has shown encouraging results in preclinical settings, including improved histological organization, reduced inflammatory infiltration, and enhanced tissue integration. These findings provide a critical foundation for the development of future clinical strategies aimed at biologically augmenting labral repair in the shoulder.

In the following section, we examine the most recent advances in hydrogel technologies applied to labral regeneration. We focus on translational in vitro and animal model studies that evaluate the efficacy of hydrogel-based systems functionalized with biologic agents, with a particular emphasis on applications relevant to glenoid labral injury. This section aims to establish the rationale, summarize key findings, and highlight the therapeutic potential of these innovative approaches in improving patient outcomes.

### Hydrogels in Labral Repair Models

Cartilage repair and regeneration of labral defects using cell-laden hydrogels remain a novel area of investigation. While not specific to glenoid labral injuries, fibrocartilage regeneration using hydrogel platforms has been explored in other tendon–bone interface (TBI) models. In a study by Chen et al., a thermosensitive hydrogel derived from enzymatically digested porcine small intestinal submucosa (SIS) was developed to serve as both a scaffold and an immunomodulatory matrix (Table 2) [9]. The SIS hydrogel retained key bioactive extracellular matrix components, enabling sustained release of endogenous signaling molecules that support tissue regeneration and macrophage modulation. Unlike traditional hydrogels, the SIS formulation remained injectable at room temperature and underwent rapid gelation at body temperature, allowing for minimally invasive delivery and in situ conformability to irregular defects. This gel also demonstrated robust biocompatibility and mechanical stability in vivo. When loaded with human urine-derived stem cells (hUSCs) genetically modified to overexpress basic fibroblast growth factor (bFGF), the SIS hydrogel promoted both immune regulation and fibrocartilage formation. Specifically, the SIS matrix facilitated polarization of macrophages toward an M2 reparative phenotype, while bFGF enhanced the proliferation and chondrogenic differentiation of hUSCs. Histological analysis with H&E and Alcian blue staining showed early fibrocartilage formation as soon as four weeks post-implantation [9]. The dual contribution of bFGF-driven differentiation and SIS-mediated immunomodulation highlights the potential of bioactive, thermoresponsive, and cell-laden hydrogels in supporting cartilage regeneration at complex musculoskeletal interfaces. By simultaneously enhancing the intrinsic chondrogenic capacity of stem cells and shaping the local immune response toward a pro-regenerative phenotype, this combined approach addresses two major barriers in fibrocartilage repair: limited cellular differentiation and chronic inflammation. The SIS hydrogel’s thermosensitive properties further enable minimally invasive delivery and conformal integration into irregular defects, making it a versatile platform for tissue engineering applications.

Together, these features underscore the great potential value in integrating biochemical, cellular, and immunological strategies into a single therapeutic construct for more effective and functional cartilage regeneration [10]. In addition to their biologic function, these hydrogels demonstrate mechanical characteristics that support labral regeneration. Thermoresponsive and adhesive-based gels are capable of maintaining positional stability in vivo, resisting displacement under physiologic loading, and conforming to irregular defect geometries through in situ gelation. Although most preclinical studies do not report specific mechanical values, these systems are designed to balance injectability, structural integrity, and bioactivity: three essential properties for successful integration and regeneration in the dynamic shoulder joint environment. These characteristics are particularly advantageous for glenoid labral applications, where irregular tear geometries, dynamic mechanical loading, and limited surgical access necessitate materials that can conform in situ and maintain function under stress.

The regenerative potential of the human glenoid labrum was investigated using human cadaveric models in the past. It has been understood that poor vascularity is the main factor that limits the regenerative capacity of cartilages [22]. Hoang et al. [92] found that a greater vascular area was associated with increased proliferative activities and progenitor cell quantity. Glenoid labral regions with higher proliferative activities were primarily identified using the Ki-67 antibody, serving as a marker for proliferative cells. The glenoid labrum was divided into four regions. The inferior glenoid labrum was found to have the highest vascularity and progenitor cell density and corresponded with the lowest reoperation rates, while, as previously described, the superior labrum had poor vascularity, as well as the lowest total cell density and Ki-67 positive cell density, indicating poor activity of proliferative cells such as fibrochondrocytes. The superior glenoid labrum was also found to have the densest extracellular matrix, suggesting that supporting cells may face difficulty in migration. This highlights the need for treatments that can enhance regional vascularity, such as the delivery of PDGF or vascular endothelial growth factor (VEGF) via hydrogels [92]. Although limited by a small sample size of only three cadaveric glenoid labra, the data obtained in this study support the notion that, similar to surgical interventions, hydrogel-based therapies should be selected and tailored according to the anatomic location of the glenoid labral injury. These regional differences in vascularity and cellular activity suggest that future hydrogel strategies could benefit from spatially targeted delivery of angiogenic and mitogenic factors, tailored to the specific anatomic zone of the labral injury.

**Figure 4:**
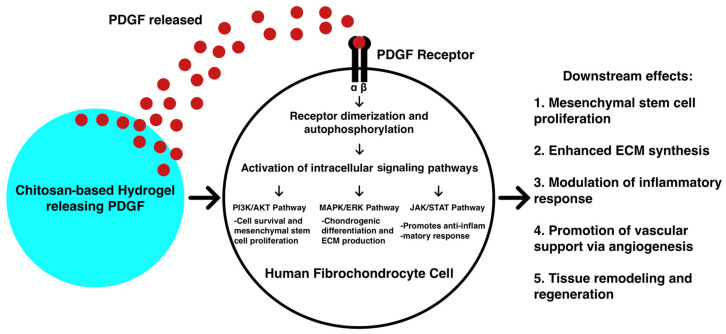
Theoretical mechanism by which PDGF-enriched chitosan-based hydrogel enhances cartilage regeneration in fibrochondrocytes.

Li et al. [93] similarly studied cartilage regeneration of the acetabular labrum, which shares structural and functional characteristics with the glenoid labrum, as both are fibrocartilaginous tissues that stabilize ball-and-socket joints. Ex vivo labra from human donors who had undergone joint replacement procedures were used to simulate labral tears. A PDGF-loaded bioadhesive was shown to recruit more labral cells and stimulate greater extracellular matrix (ECM) production than both the adhesive-only group and a control group treated with media alone (Figure 4). The study also found that in the chitosan-based gel, PDGF exhibited a faster release profile due to electrostatic repulsion between the positively charged gel and PDGF molecules. While the adhesive-only group also showed signs of labral regeneration, the effects were less pronounced than those observed with the PDGF-releasing adhesive [93]. However, the authors noted that the chitosan-based hydrogel was limited by its insufficient mechanical strength. They proposed incorporating nanocellulose to form double-crosslinked networks, suggesting that improving the mechanical integrity of the gel could significantly enhance acetabular labrum repair and, translationally, glenoid labrum healing and cartilage regeneration. Given the distinct differences in joint mechanics between the shoulder and hip, further work is needed to optimize hydrogel formulations that can withstand the repetitive shear forces and high mobility of the glenohumeral joint while maintaining cellular viability and bioactivity.

In their rat glenoid labral tear model, Co et al. [94] compared three groups—injured labra without treatment (control), injured labra treated with bioadhesive only, and injured labra treated with PDGF-releasing adhesive—thereby demonstrating that the PDGF-enhanced treatment yielded the most robust progenitor cell recruitment and tissue healing. In comparing injured versus healthy glenoid labra, the authors used CD44+ and CD73+ markers to detect the presence of mesenchymal progenitor cells. They found that injured labra contained more progenitor cells than uninjured, healthy labra and that injured labra treated with the PDGF-releasing adhesive exhibited the highest number of progenitor cells [94]. These findings suggest that progenitor cells may begin migrating from the joint capsule to sites of labral injury. The results also support the well-established concept that PDGF possesses strong chemotactic activity for mesenchymal progenitor cells, particularly during wound healing and tissue regeneration.

Beyond progenitor cell recruitment, Co et al. also demonstrated that the PDGF-releasing adhesive significantly attenuated inflammatory responses, as evidenced by reduced infiltration of CD11b+ immune cells and decreased expression of pro-inflammatory cytokines such as IL-1β and MMP-13 [94]. These effects may contribute to the preservation of labral tissue and mitigation of degeneration. Notably, both the adhesive-only and PDGF-adhesive groups exhibited increased extracellular matrix deposition, including collagen and glycosaminoglycans (GAGs), with the PDGF-treated group showing the greatest level of enhancement. This suggests that growth factor delivery not only modulates the inflammatory microenvironment but also actively promotes tissue remodeling and regeneration. Taken together, these results underscore the importance of designing hydrogel systems that simultaneously address three key barriers to effective glenoid labral repair: poor intrinsic vascularity, chronic inflammation, and insufficient mechanical stability under joint motion. While these early studies demonstrate promising regenerative outcomes in preclinical models, several important hurdles remain for clinical translation. In addition, the dynamic mechanical environment of the glenohumeral joint presents unique challenges for the retention, durability, and functional integration of hydrogel-based implants. Key hurdles also include ensuring reproducible manufacturing of biologic-laden hydrogels, meeting regulatory safety standards, and validating long-term efficacy in load-bearing human joints. Future research may explore synergistic combinations of pro-angiogenic, chondrogenic, and immunomodulatory agents, as well as advanced delivery platforms that enable zonal targeting within the complex architecture of the glenoid labrum.

The current animal models to study labral pathology may have limitations: A comparative study was conducted by Como et al. [95] to identify the animal model with a shoulder labrum resembling human anatomy the closest. After conducting anatomic and histological evaluations, it was found that common animal models including rats, dogs, goats, rabbits, and pigs do not possess a distinct glenoid labrum [95], hence complicating the effort to assess the effect of various therapeutic options. This finding challenges earlier presumptions about their suitability for modeling labral injury. Consequently, evaluating therapeutic interventions in such models may be problematic and underscores the need to develop better animal models, perhaps in nonhuman primates, and, ultimately, to pursue clinical trials to validate the use of hydrogels in labral treatment.

## 4. Conclusions

Hydrogels have demonstrated considerable promise in the regeneration of cartilage and healing of soft tissue injuries, including early insights into applications of glenoid labral tears. Preclinical models and translational studies suggest that hydrogels, particularly those delivering growth factors such as PDGF, VEGF, and FGF, can enhance labral repair by supporting extracellular matrix production, modulating inflammatory responses, and recruiting progenitor cells. However, despite these encouraging findings, the body of research specifically addressing hydrogel-based glenoid labral repair remains limited. Most studies to date have relied on small-animal models or ex vivo tissues, and clinical translation is still in the early stages. Given the complex biomechanical environment of the shoulder joint and the unique challenges associated with labral healing, further high-quality research, including large-animal studies and eventual human clinical trials, is critically needed. Optimized hydrogel formulations and delivery systems must be developed that can integrate effectively with current surgical techniques while accommodating the regional vascular and biomechanical variations of the glenoid labrum. Personalized approaches that tailor hydrogel composition and growth factor delivery to individual injury characteristics may further enhance clinical outcomes. Continued innovation in this field will be essential to enable safe, effective, and widespread clinical adoption of hydrogel-based therapies for glenoid labral repair.

## Figures and Tables

**Table 1:** Quantitative comparison of natural, synthetic, and hybrid hydrogels used in cartilage regeneration, highlighting representative materials, fabrication methods, degradation times, cell viability, and key performance mechanisms. Data are compiled from published studies [58,59,60,61,62,63,64,65,66,67,68,69,70,71,72,73,74,75,76]. Abbreviations: PCL, polycaprolactone; PVA, polyvinyl alcohol; PEG, polyethylene glycol; ECM, extracellular matrix.

Hydrogel Type	Example Materials	Fabrication/Crosslinking Method	Degradation Time	Cell Viability (%)	Mechanism of Performance	Ref.
Natural	Chitosan + gelatin	Chemical (citric acid) Physical (ionic and H-bond)	1–3 weeks (unmodified), 4–8 weeks (with crosslinking)	>85–95% in 1–2 weeks	High biocompatibility, low cytotoxicity Support chondrocyte adhesion and proliferation Promote extracellular matrix synthesis Degradation rate and stability tunable by polymer type/crosslinking Mimic native ECM for tissue integration	[58,59,60]
Synthetic	PEG, PCL, and PVA	UV photopolymerization, Michael addition, and thiol-ene Freeze–thaw and chemical crosslinking Copolymer/blend and UV crosslinking	~1–12 months (tunable by chemistry and crosslinking)	>85–95% with appropriate functionalization; lower without adhesion ligand modification	Highly tunable mechanical and degradation properties High structural stability compared to natural hydrogels Often bioinert; requires functionalization for cell adhesion Controlled network density enables precise growth factor release	[65,66,67,68,69]
Hybrid	PEG–PCL, PVA–alginate, gelatin–PCL, PEG–gelatin, and chitosan–PCL	Physical + chemical crosslinking (e.g., freeze–thaw, UV, enzymatic, and citric acid)	Weeks to months, tunable by polymer ratio and crosslinking density	>90–95%; natural component supports adhesion and proliferation; synthetic component maintains stability	Combine bioactivity of natural polymers (adhesion sites, ECM mimicry) with mechanical strength of synthetics Tunable degradation by adjusting polymer ratio and crosslinking density Dual crosslinking (physical + chemical) improves stability and functionality Support chondrocyte adhesion, proliferation, and extracellular matrix synthesis	[70,71,72,73,74,75,76]

## Data Availability

No new data were created or analyzed in this study. Data sharing is not applicable to this article.
